# A year of change for *Journal of Molecular Cell Biology*

**DOI:** 10.1093/jmcb/mjaa001

**Published:** 2020-02-17

**Affiliations:** Institute of Biochemistry and Cell Biology, Shanghai Institutes for Biological Sciences, Chinese Academy of Sciences, Shanghai 200031, China

In recent years, the scientific publishing world has been significantly changed from focusing on impact factor to encouraging scientific merit and from subscriptional access to open access (Wu, 2018). *Journal of Molecular Cell Biology* (*JMCB*), which was launched in October 2009, started a new journey following the new routes in the scientific publishing world from the beginning of 2019. Here, I would like to go through what *JMCB* has fulfilled in the first year of change.

As an academic journal, *JMCB* devotes itself to expressing the appreciation on the important scientific discoveries in the past and bringing the new development based on these discoveries for the future. In 2019, *JMCB* focused on the famous tumor suppressor gene *p53*, which was discovered 40 years ago. A memorial symposium with the theme ‘The Legend of p53 vs. Cancer’ was hosted by *JMCB* in May 2019 in Hangzhou, China (Wu, 2019; Zhang et al., 2019). In addition, two special issues on ‘p53: updates on mechanisms, biology and therapy’ (Lane and Verma, 2019a, b) and one memorial issue for celebrating the 40th anniversary of p53 discovery (Lu, 2019) were published. It is worth mentioning that a number of hard core researchers, many of whom are pioneering and world-class scientists in the fields of p53, epigenetics, and cancer, contributed to this series of events. Remarkably, Dr Arnold J. Levine, one of the p53 discoverers, delivered his perspective on p53 research after four decades, entitled ‘The many faces of p53: something for everyone’ (Levine, 2019).

*JMCB* also recognizes that science benefits from openness. Since January 2019, *JMCB* has been transformed from a traditional subscription journal to a fully open access one. This transformation has significantly increased the accessibility, evidenced by the fact that the total downloads of *JMCB* publications per month in 2019 increased by more than 70%, compared with that in 2015–2018. In addition, *JMCB* allows advance posting on preprint servers, such as bioRxiv, prior to publication to help authors share their work as early as possible and receive feedback from a wider audience. We believe that open access publication of *JMCB* provides a more direct and efficient way for science communication, and in turn, the journal will benefit from an open scientific community.

The new decade is coming! While the ecosystem of scientific publishing world is still in course of reconstruction, *JMCB* will do its best in this changing world and provide better service for the community.
